# The presence of autistic traits might explain the relationship between sensory sensitivity and eating disturbances in a sample of young adults referring to a mental health clinic.

**DOI:** 10.1192/j.eurpsy.2024.484

**Published:** 2024-08-27

**Authors:** V. Nistico’, G. Ingrosso, F. Lombardi, E. Chiudinelli, R. Faggioli, A. Bertani, O. Gambini, B. Demartini

**Affiliations:** ^1^Health Science Department, University of Milan; ^2^Department of Psychology, University of Milan Bicocca; ^3^Aldo Ravelli Research Center for Neurotechnology and Experimental Brain Therapeutics, University of Milan; ^4^UO Psichiatria 51 e 52; ^5^Centro Giovani Ettore Ponti, ASST Santi Paolo e Carlo, Presidio San Paolo, Milan, Italy

## Abstract

**Introduction:**

The relationship between autistic traits and eating disturbances, although gaining considerably more attention in the last decades, is still unclear. Most of the studies up to date were conducted on individuals with a full diagnosis of Autism Spectrum Disorders (ASD) and/or of Eating Disorders (ED). One of the common features reported in both conditions is the alteration of sensory sensitivity, which is, in both cases, widely discussed in the literature, but mostly in the pediatric age.

**Objectives:**

To investigate the association between sensory sensitivity, autistic traits, and eating disorders symptomatology in a group of young adults (18-24) who were referred, for the first time, to a mental health outpatient clinic.

**Methods:**

259 patients completed: the Eating Attitude Test (EAT-26), the Autism Quotient (AQ), the Ritvo Autism Asperger Diagnostic Scale-Revised (RAADS-R), the Sensory Perception Quotient - Short Form 35 item (SPQ-SF35) and the Swedish Eating Assessment for Autism Spectrum Disorders (SWEAA), which investigates specific eating behaviour related to autism.

**Results:**

23.55% participants scored above the cut-off at the EAT-26, suggesting that they should be assessed for the presence of an eating disorder by a specialized clinician. The RAADS-R explained a great proportion of variance in the relationship between sensory sensitivity and both the SWEAA (Total Score and subscales) and the EAT-26 (Total Scores and subscales).

**Conclusions:**

Our study revealed a substantial prevalence of potential eating disorders among young adults in our sample, with nearly one-fourth of participants surpassing the EAT-26 cutoff score. Additionally, we observed a noteworthy association between the presence of autistic traits and not only autistic-like eating behaviors but also a broader spectrum of eating disorder symptoms; this relationship was found in a cohort of young adult patients seeking clinical attention due to generalized distress, prior to receiving specific diagnoses of Autism Spectrum Disorder (ASD) or Eating Disorders (ED). These findings give rise to several intriguing inquiries. Could the existence of autistic traits, even when subthreshold, function as a mediator between alterations in sensory sensitivity and the emergence of maladaptive eating behaviors? Furthermore, if these traits exist at subthreshold levels, might they manifest in various psychiatric conditions, distinct from traditional categorizations, during episodes of acute distress? What potential precipitating factors should be considered in such cases?

**Disclosure of Interest:**

None Declared

